# Comparison of isocaloric very low carbohydrate/high saturated fat and high carbohydrate/low saturated fat diets on body composition and cardiovascular risk

**DOI:** 10.1186/1743-7075-3-7

**Published:** 2006-01-11

**Authors:** Manny Noakes, Paul R Foster, Jennifer B Keogh, Anthony P James, John C Mamo, Peter M Clifton

**Affiliations:** 1 CSIRO Health Sciences and Nutrition, Adelaide, Australia; 2 Curtin University, Western Australia

## Abstract

**Background:**

It is speculated that high saturated fat very low carbohydrate diets (VLCARB) have adverse effects on cardiovascular risk but evidence for this in controlled studies is lacking. The objective of this study was to compare, under isocaloric conditions, the effects of a VLCARB to 2 low saturated fat high carbohydrate diets on body composition and cardiovascular risk.

**Methods:**

Eighty three subjects, 48 ± 8 y, total cholesterol 5.9 ± 1.0 mmol/L, BMI 33 ± 3 kg/m^2 ^were randomly allocated to one of 3 isocaloric weight loss diets (6 MJ) for 8 weeks and on the same diets in energy balance for 4 weeks: Very Low Fat (VLF) (CHO:Fat:Protein; %SF = 70:10:20; 3%), High Unsaturated Fat (HUF) = (50:30:20; 6%), VLCARB (4:61:35; 20%)

**Results:**

Percent fat mass loss was not different between diets VLCARB -4.5 ± 0.5, VLF-4.0 ± 0.5, HUF -4.4 ± 0.6 kg). Lean mass loss was 32-31% on VLCARB and VLF compared to HUF (21%) (P < 0.05). LDL-C increased significantly only on VLCARB by 7% (p < 0.001 compared with the other diets) but apoB was unchanged on this diet and HDL-C increased relative to the other 2 diets. Triacylglycerol was lowered by 0.73 ± 0.12 mmol/L on VLCARB compared to -0.15 ± 0.07 mmol/L on HUF and -0.06 ± 0.13 mmol/L on VLF (P < 0.001). Plasma homocysteine increased 6.6% only on VLCARB (P = 0.026). VLCARB lowered fasting insulin 33% compared to a 19% fall on HUF and no change on VLF (P < 0.001). The VLCARB meal also provoked significantly lower post prandial glucose and insulin responses than the VLF and HUF meals. All diets decreased fasting glucose, blood pressure and CRP (P < 0.05).

**Conclusion:**

Isocaloric VLCARB results in similar fat loss than diets low in saturated fat, but are more effective in improving triacylglycerols, HDL-C, fasting and post prandial glucose and insulin concentrations. VLCARB may be useful in the short-term management of subjects with insulin resistance and hypertriacylglycerolemia.

## Background

Obesity, particularly abdominal obesity, contributes substantially and directly to cardiovascular risk as well as exacerbating associated risk factors such as dyslipidaemia, hypertension and diabetes [1,2]. Although weight loss has been shown to reverse many of these associated risk factors[3] defining optimum long-term eating patterns for weight loss is important in order to optimize risk factor reduction, given that the evidence for the benefit on cardiovascular mortality of weight reduction alone is conflicting [4,5]. The resurgence of interest in diets promoting low carbohydrate intake or high protein intake is occurring at a time where there is mounting evidence that high intakes of refined carbohydrates have paralleled the development of obesity and type 2 diabetes [6]). There have been a number of studies examining the effect of very low carbohydrate diets using an ad libitum approach as per the Atkins diet and they have demonstrated superior weight loss on very low carbohydrate diets compared to low fat high carbohydrate diets over a 6 month period [7-13]. Although this seems to be consistent with the notion that very low carbohydrate diets have a metabolic advantage[11,14], the design of these studies was not planned to test this hypothesis as they assessed very low carbohydrate diets on an ad libitum basis (thereby not controlled for kilojoule intake) and assessed cardiovascular risk factors during energy restriction which may exaggerate the net effects of weight loss on that diet composition.

The aim of this study was therefore to evaluate under isocaloric conditions a very low carbohydrate dietary pattern (<20 g carbohydrate/day) compared to a very low fat diet or a low saturated fat high unsaturated fat dietary pattern. Endpoints were body composition and a range of conventional and novel cardiovascular risk markers.

## Methods

### Subjects and design

**Figure 1:**
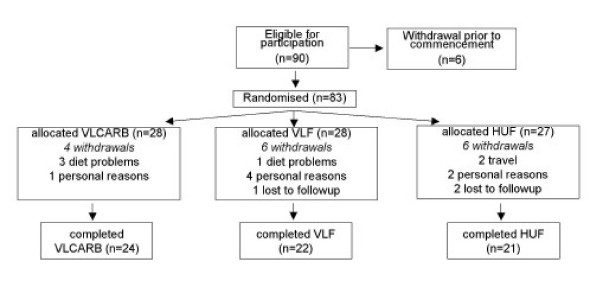
Schematic representation of randomization.

**Figure 2:**
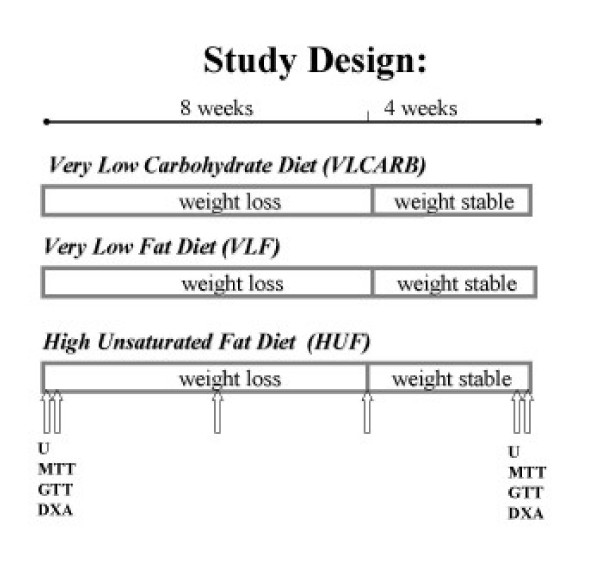
**Schematic representation of study design**. U = 24 hour urine. MTT = meal tolerance test. GTT = glucose tolerance test. DXA = Dual Xray Absorptiometry. ↑ = blood sample. VLCARB = very low carbohydrate diet (n = 24) VLF = very low fat diet (n = 22) HUF = high unsaturated fat (n = 21).

Ninety subjects with at least one cardiovascular risk factor in addition to a BMI>28 were recruited by public advertisement to participate in a clinical trial of 12 weeks duration. Seven subjects withdrew before randomization and a further 16 withdrew during the study leaving a total of sixty seven subjects that completed the study (Figure 1). Subjects were matched on the basis of age, gender, BMI and randomly allocated to one of 3 dietary intervention groups (Table 1). There was an intensive weight loss period of 8 weeks and a weight maintenance period of 4 weeks duration. The protocol and potential risks and benefits of the study were fully explained to each subject before they provided written informed consent. A schematic representation of study design is shown in Figure 2. All experimental procedures were approved by the Human Ethics Committees of the Commonwealth Scientific Industrial Research Organisation (CSIRO).

**Table 1:** Subject characteristics at baseline^1^

	VLCARB	VLF	HUF
males/females	4/20	5/17	3/18
BMI kg/m^2^	32.5 ± 3.1	32.6 ± 4.0	33.4 ± 3.6
AGE y	48.4 ± 8.0	50.7 ± 10.3	46.1 ± 9.5
Total Cholesterol mmol/L	5.8 ± 1.0	5.6 ± 1.1	6.0 ± 1.1
LDL-C mmol/L	3.8 ± 0.8	3.6 ± 1.1	4.0 ± 1.1
HDL-C mmol/L	1.2 ± 0.2	1.3 ± 0.3	1.2 ± 0.2
Triacylglycerols mmol/L	1.8 ± 0.9	1.5 ± 0.6	1.6 ± 0.5

**^1 ^Data are Mean ± SD**. VLCARB = very low carbohydrate diet (n = 24) VLF = very low fat diet (n = 22) HUF = high unsaturated fat (n = 21)

### Dietary intervention

The diets were designed to be isocaloric and 30% energy restricted (~6000 kJ) for 8 weeks, followed by 4 weeks on the same macronutrient proportions but maintaining energy balance. Energy requirements of individuals were calculated and 30% energy restriction calculated accordingly The planned macronutrient profiles of the treatment diets were as follows: Very Low Fat (VLF) (CHO:Fat:Protein; %SF = 70:10:20; 3%), High Unsaturated Fat (HUF) = (50:30:20; 6%), VLCARB (4:61:35; 20%). Templates for the dietary patterns were developed for 5.5 MJ, 6 MJ, 7 MJ and 8 MJ and these served as a basis for minor individual adjustments.

Key foods for each diet (2200 kilojoules or 36% of total energy) representative of the diets' macronutrient profile were supplied every 2 weeks for the 12 wk study. Foods provided were uncooked but pre-weighed to facilitate compliance. The dietary pattern was structured to include specific daily quantities of foods to ensure the correct macronutrient and energy requirements (Table 2A). These foods were listed in a checklist which subjects completed on a daily basis. Detailed dietary instruction, meal planning and recipe information was provided at baseline and every 2 weeks by a qualified dietitian. Checklists of prescribed foods and additional foods were checked, and 3 d weighed food records were collected every 2 wks to assess dietary compliance up to the 12 wk time point. At week 8, adjustment of energy intake was titrated upwards to restore the daily energy deficit based on the calculated daily deficit assessed from the previous 4 wks weight loss. Standard meal plans for each diet were developed for a range of energy levels in 1000 kJ increments which served as the basis for the revised energy balance prescription. Subjects were counselled by a dietitian on the dietary protocol and on how to keep dietary-intake checklists for all foods consumed each day over the study duration. The subjects' body weight and dietary-intake checklist were monitored every 2 weeks and dietary adjustments were made if necessary. Three consecutive days (one weekend and 2 weekdays) of the checklists from each 2-week period were analyzed by "Diet/1 Nutrient Calculation" software (Xyris Software 1998, Highgate Hill, Australia), a computerized database of Australian foods. Recipes were entered as proportions of the original ingredients. The database had been extensively modified by our group to add new foods and recipes. A questionnaire with numerical rating scale was used to assess diet acceptability on a range of parameters including palatability, ease of adherence, effect on hunger, fullness, nausea and desire to eat, and cravings.

**Table 2A:** Food profile of treatment diets

***VLCARB***		***VLF***		***HUF***	
Cheese, full fat	70 g	High fibre cereal	40 g	High fibre cereal	32 g
Milk, full fat	125 g	Bread, wholegrain	105 g	Bread, wholegrain	70 g
Lean meat, chicken	350 g	Low fat biscuits	60 g	Milk, skim	300 g
Eggs	2	Milk, skim	250 g	Cheese full fat	20 g 2/week
Very low carbohydrate vegetables	2 cups	Cheese low fat	20 g	Yoghurt, skim	200 g × 3/week
Almonds	50 g	Rice or pasta, dry	50 g	Pasta or rice, dry	100 g × 4/week
Butter	20 g	Fresh fruit	300 g	Nuts, mixed	20 g
		Dried fruit	50 g	Salad vegetables	100 g
		Lean meat, chicken	100 g	Fresh fruit	300 g
		Salad vegetables	100 g	Pulses, cooked	100 g × 2/week
		Low carbohydrate vegetables	2 cups	Lean meat, chicken,	150 g 5/week
				Fish	150 g/week
				Sardines	3 whole/week
				Tuna	50 g × 2/week
				Low carbohydrate vegetables	1.5 cups
				Potato	1 × 3/week
				Unsaturated oil or margarine	25 g

### Clinical and biochemical analyses

Blood samples were taken according to the schedule as per Figure 2. Venous blood samples were taken in the morning after an overnight fast for determination of plasma glucose, insulin, ketones and lipid concentrations. Fasting serum was collected, and stored at -20°C until the end of the study. All lipid assays were performed in a single run at the end of the 12-week study on a Roche Cobas-Bio centrifugal Analyser using standard Roche enzymatic kits. HDL cholesterol was measured after PEG 6000 precipitation of apoB containing lipoproteins. Fasting lipids were taken on two consecutive days and then values averaged at each of the timepoints. Coefficients of variance (CV) for all assays were less than 5% with the exception of insulin for which the CV was 9.75%. Total apoB (B100 and B48) was measured by immunoturbidimetry using Roche antisera. Apo B48 levels were determined directly in serum using a Western Blotting procedure as previously described by James et al [15]. Following visualisation using enhanced chemiluminescence (Amersham, Little Chalfont, UK) apo B48 bands were identified and quantified by densitometry against purified apo B48 protein of known mass using NIH Image (version 1.6.3). The mean intra- and inter-assay CV for apo B48 were each less than 4% Plasma levels of ketones were assayed using an enzymatic method using the principle that β hydroxybutyrate in the presence of NAD is converted to acetoacetate and NADH by β-hydroxybutyrate dehydrogenase. The NADH produced was quantified spectrophotometrically on a Cobas-Bio centrifugal Analyser measured by absorbance at 340 nm.

At weeks 0 and 12, a single venous blood sample was taken for the determination of homocysteine, folate and B12 which were measured in a certified commercial laboratory (Institute of Medical and Veterinary Science, Adelaide, South Australia). Serum CRP concentrations (CV 3.5%) were measured in duplicate at baseline and at week 12 with an ultrasensitive ELISA (Alpha Diagnostica). Serum insulin was measured by radioimmunoassay (Pharmacia & Upjohn Diagnostics AB, Uppsala, Sweden) while CRP was measured by immunoturbidimetry using Roche antisera.

A 24-hour urine sample was collected for the assessment of the urea/creatinine ratio, calcium, sodium and potassium excretion, as well as deoxy-pyridinoline/creatinine and pyridinoline/creatinine ratios (biomarkers of bone turnover) were also assessed from the 24-hour urine sample at weeks 0 and 12. Urine samples were frozen, and urea and creatinine was measured in one run at the end of the study using a Hitachi auto analyzer (Roche, Indianapolis, USA). Urinary pyridinium crosslinks (markers of bone turnover) were measured using HPLC. Urine samples were measured at the Institute of Medical and Veterinary Science, Adelaide, South Australia) for calcium, phosphate and sodium using proprietary techniques on the Olympus AU5400 chemistry analyzer (Japan).

Also at weeks 0 and 12, measurements of body composition and a 75 g oral glucose tolerance test (GTT) and on a subsequent day a 3-hour meal tolerance test (MTT) using meals that were representative of the diet to which the subjects were assigned (Table 2B), were performed. Venous blood samples for the determination of glucose, insulin, free fatty acid concentrations were taken prior to consuming the test meals as well as at 30, 60, 120 and 180 minutes after the meal.

**Table 2B:** Foods and nutrient profiles provided in Meal Tolerance Test (MTT)

**VLCARB**	**Amount(g)**	**Energy**	**Protein (g)**	**Fat (g)**	**Carbohydrate (g)**
Cheese	40	676	10	14	0
Corned beef	60	247	11	2	0
Ham	60	272	11	2	0
Egg	50	316	7	5	0
Whole milk	50	136	2	2	2
Almonds	20	486	4	11	1
Salad	50	26			1
**total**		**2159**	**45**	**36**	**4**
**% energy**			**36%**	**61%**	**3%**

**VLF**					
Bread wholemeal	100	939	10	3	39
Cheese reduced fat	10	137	3	2	0
Corned beef	10	41	2	0	0
Ham	10	45	2	0	0
Skim milk	150	276	7	0	10
Fruit bar	1 bar	548	1	1	29
Sultanas	15	192	0	0	11
**total**		**2178**	**25**	**7**	**89**
**% energy**			**20%**	**13%**	**67%**

**HUF**					
Bread wholemeal	110	1033	11	3	43
Margarine polyunsaturated	12	359	0	10	0
Corned beef	20	82	4	1	0
Salmon	20	146	4	2	0
Yoghurt	200	410	10	0	12
Baked beans	40	114	2	0	4
Salad	50	26	0	0	1
**total**		**2169**	**32**	**16**	**60**

**% energy**			**26%**	**28%**	**46%**

VLCARB = very low carbohydrate diet (n = 24) VLF = very low fat diet (n = 22) HUF = high unsaturated fat (n = 21)

The homeostatic model assessment (HOMA) was used as a surrogate measure of insulin sensitivity and was calculated as [fasting serum insulin (mU/L) × fasting plasma glucose (mmol/L)/22.5] ([16]). Total glucose, insulin and area under the curve during the 3-hour GTT and MTT was calculated geometrically using the trapezoidal rule [17].

Body composition was determined by whole body DEXA using a Norland Densitometer XR36; (Norland Medical Systems, Fort Atkinson, Wisconsin, USA; CV of 2.3 ± 0.7% for total body fat mass and 2.1 ± 0.4% for lean mass) at baseline (prior to commencement) and at 12 weeks. Blood pressure was measured using an HDI/Pulsewave™ instrument (Hypertension Diagnostics inc. Minnesota, USA).

### Statistical analysis

Statistical analysis was performed using SPSS for Windows 10.0 software (SPSS Inc, Chicago, USA). Baseline measurements were assessed using two-factor ANOVA with diet and gender as the fixed factors. The effect of the diet intervention was assessed using repeated-measures ANOVA; for each dependent variable, the measurements at weeks 0, 4, 8, and 12 are the within-subject factor (i.e. time) and diet and gender are the between-subject factors. Week 0 and 12 response curves following the GTT and test meals were compared using repeated measures ANOVA with week and blood sampling time (or AUC) as the within-subject factors and diet as the between-subject factors. When significant time-by-diet effects were found, post hoc sub-group analysis was performed using Tukey's test. The study had 80% power (α = 0.05) to detect differences between dietary groups of 3.6 kg in body weight, 0.9 kg in lean and fat mass, 3 mU/L in fasting insulin and 0.2 mmol/L in LDL-cholesterol. Significance was set at P < 0.05. All data except baseline characteristics are presented as means ± SEM, unless stated otherwise.

## Results

### Dietary compliance

**Figure 3:**
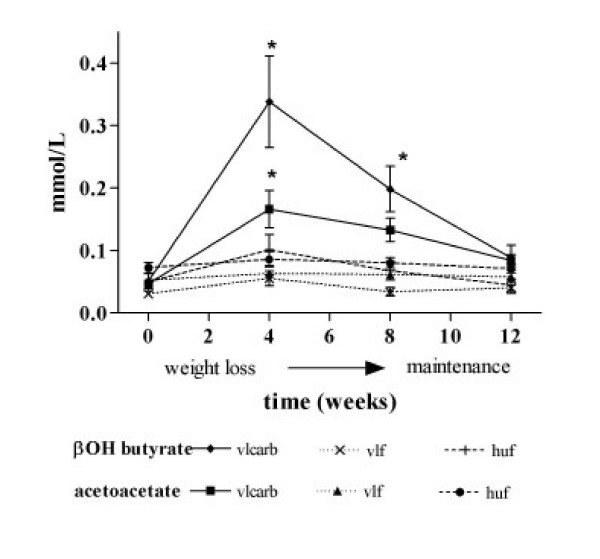
**Plasma ketones during weight loss and weight maintenance, according to dietary treatment^1^**. ^1^mean ± SEM. * denotes a significant difference of VLCARB from VLF and HUF (p < 0.05) by one way ANOVA at each time point for main effect of diet (p < 0.05) and post hoc Tukeys test to detect differences (p < 0.05). VLCARB = very low carbohydrate diet (n = 24) VLF = very low fat diet (n = 22) HUF = high unsaturated fat (n = 21)

Reported dietary intake was consistent with the prescribed dietary treatments (Table 3). Compliance to dietary treatment was also confirmed by a change in plasma ketones between diets. VLCARB produced higher plasma levels of ketones (β hydroxybutyrate and acetoacetate) than the VLF or HUF diet treatments (P < 0.01), indicating adherence to a very low carbohydrate intake during the study (Figure 3). Despite continued apparent compliance to the diet plasma ketones declined with time.

**Table 3:** Nutrient intake by dietary treatment during weight loss and weight maintenance assessed using weighed food records^12^

	**VLCARB**		**VLF**		**HUF**	
**Nutrient**	**Weight loss**	**Maintenance**	**Weight loss**	**Maintenance**	**Weight loss**	**Maintenance**

**Energy (kJ)**	6193 (± 82)	7706 (± 167)	6061 (± 168)	7000 (± 333)	5996 (± 88)	7659 (± 201)
**% energy protein^3^**	33.1 (± 0.85)	30.5 (± 0.91)	19.9 (± 0.33)	20.3 (± 0.55)	22.6 (± 0.46)	21.4 (± 0.51)
**% energy fat^3^**	55.1 (± 1.96)	54.3 (± 2.53)	11.7 (± 0.32)	12.5 (± 0.59)	27.4 (± 0.84)	28.0 (± 0.88)
**% energy carbohydrate^3^**	8.8 (± 2.71)	12.4 (± 3.38)	67.7 (± 0.60)	66.0 (± 0.92)	47.9 (± 0.83)	48.7 (± 1.07)
**% energy saturated fat^4^**	17.6 (± 0.77)	17.7 (± 1.01)	4.5 (± 0.16)	5.1 (± 0.30)	5.4 (± 0.18)	6.0 (± 0.32)
**% energy MUFA^3^**	27.0 (± 1.16)	26.2 (± 1.41)	3.3 (± 0.11)	3.6 (± 0.21)	12.0 (± 0.48)	12.3 (± 0.51)
**% energy PUFA^3^**	6.3 (± 0.13)	6.5 (± 0.28)	1.7 (± 0.03)	1.8 (± 0.16)	7.6 (± 0.29)	7.2 (± 0.30)
**Calcium (mg)^5^**	959 ± 14	1297 ± 58	867 ± 32	1079 ± 55	969 ± 19	1169 ± 43

**^1 ^m**ean ± SEM, VLCARB = very low carbohydrate (n = 24) VLF= very low fat (n = 22) HUF = high unsaturated fat (n = 21)

**MUFA **= monounsaturated fat, **PUFA **= polyunsaturated fat

^2 ^Three days (2 week days and 1 weekend day) of dietary data were analysed at weeks 2, 4, 6 and 8 during the weight loss period and at weeks 10 and 12 during the maintenance period. No significant differences were found between the four diet records in the weight loss period or between the two records for the maintenance period, so data for recordings in each period were averaged.

^3 ^Significant main effect of diet using one way ANOVA with all diets significantly different from each other (P < 0.01)

^4 ^Significant main effect of diet using one way ANOVA; VLF vs HUF (p = 0.126) and VLCARB different to VLF and HUF (P < 0.01)

^5 ^Significant main effect of diet using one way ANOVA; VLF different to VLCARB and HUF (P < 0.05)

### Weight loss

**Figure 4:**
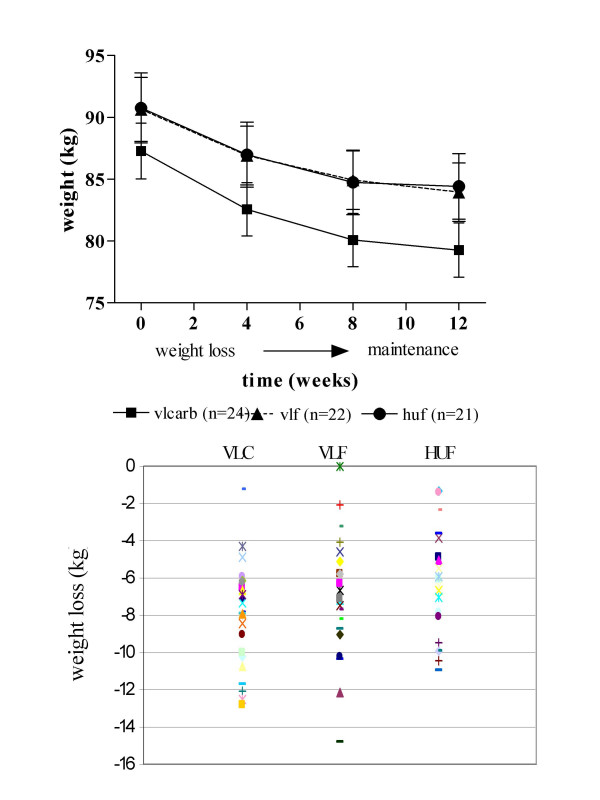
**Weight loss according to dietary treatment^1^**. ^1^mean ± SEM. VLCARB = very low carbohydrate diet (n = 24) VLF = very low fat diet (n = 22) HUF = high unsaturated fat (n = 21). There were no significant differences in absolute weight loss according to dietary treatment.

Each treatment group reduced weight over the 8 wk energy restriction period and maintained this weight during the subsequent 4 wk period (Figure 4). There were no significant differences in absolute weight loss by diet composition, with a net weight loss of 8.0 ± 0.6 kg (n = 24), 6.7 ± 0.7 kg (n = 22) and 6.4 ± 0.6 kg (n = 21) on the VLCARB, VLF and HUF diets respectively (P = 0.18). However, percentage change in weight from baseline differed significantly by diet (P = 0.034) with the VLCARB diet resulting in a greater weight loss of 9.2% compared to the VLF (7.3%) and HUF (7.0%). After applying Tukey's post hoc test however, only HUF remained significantly different to VLCARB (P = 0.044).

### Body composition

DEXA data indicated both the VLCARB and VLF diets resulted in significantly more lean mass loss as a proportion of weight loss (32% and 31%) compared to the HUF diet (21%) (P < 0.05) whereas the proportion of fat loss did not differ between diets (Table 4). DEXA data also indicated no significant differences in regional fat or lean mass loss between diets.

**Table 4:** Body composition changes according to dietary treatment^1^

***DIET***	***VLCARB***	***VLF***	***HUF***
Lean mass at baseline (kg)	46.5 ± 1.9	48.5 ± 2.6	46.4 ± 2.2
Lean mass after weight loss (kg)	43.9 ± 1.8	46.5 ± 2.3	45.0 ± 2.0
% change^2^	-2.6 ± 0.4^a^	-2.1 ± 0.4^a^	-1.4 ± 0.4^b^
Fat mass at baseline (kg)	37.6 ± 1.3	37.9 ± 2.2	40.7 ± 1.6
Fat mass after weight loss (kg)	33.1 ± 1.3	33.9 ± 2.2	36.3 ± 1.4
% change in fat mass	-4.5 ± 0.5^a^	-4.0 ± 0.5^a^	-4.4 ± 0.6^a^
% change in weight^3^	-8.0 ± 0.6^a^	-6.7 ± 0.7^a^	-6.4 ± 0.6^b^

^1 ^mean ± SEM

VLCARB = very low carbohydrate (n = 24) VLF = very low fat (n = 22) HUF = high unsaturated fat (n = 21)

^2 ^Significant effect of diet by one way ANOVA and screening weight as a covariate (P = 0.022).

^3 ^Significant effect of diet by one way ANOVA and screening weight as a covariate (P = 0.034).

Variables with different superscripts are significantly different by one way ANOVA with Tukey's test for post hoc analysis p < 0.05.

### Cardiovascular risk markers

There was a significant main effect of diet on LDL-C with a net increase of 0.18 ± 0.18 mmol/L on the VLCARB diet, but net decreases of 0.40 ± 0.11 mmol/L on the VLF and 0.34 ± 0.14 mmol/L on HUF (p = 0.008 unadjusted and p = 0.006 adjusted for weight loss) (Table 5). However, the effect of diet composition on apoB concentrations was not significant (p = 0.418) although concentrations declined with weight loss overall (p = 0.011). ApoB was unchanged in the VLCARB group. Diet significantly affected apoB48 (p = 0.05) but not after adjusting for weight loss (p = 0.11), increasing on VLF and decreasing on VLCARB and HUF (data not shown).

**Table 5:** Plasma lipoproteins, glucose, insulin, folate and homocysteine concentrations during the dietary interventions^1^

		***Baseline***	***Week 4***	***Week 8***	***Week 12***	***Change^3^***
**Total Cholesterol **mmol/L	VLCARB	5.92 ± 0.21	5.38 ± 0.20	5.68 ± 0.29	5.82 ± 0.26	-0.09 ± 0.20
	VLF	5.64 ± 0.23	4.83 ± 0.20	4.94 ± 0.23	5.15 ± 0.26	-0.49 ± 0.14
	HUF	6.09 ± 0.23	5.11 ± 0.23	5.27 ± 0.26	5.62 ± 0.24	-0.47 ± 0.15
**LDL Cholesterol^2 ^**mmol/L	VLCARB	3.83 ± 0.18	3.57 ± 0.21	3.89 ± 0.28	4.01 ± 0.26	0.18 ± 0.18^a^
	VLF	3.65 ± 0.22	3.05 ± 0.18	3.16 ± 0.20	3.25 ± 0.22	-0.40 ± 0.11^b^
	HUF	4.12 ± 0.24	3.38 ± 0.20	3.54 ± 0.25	3.78 ± 0.22	-0.34 ± 0.14^b^
**ApoB **g/L	VLCARB	1.01 ± 0.05		0.94 ± 0.05	1.00 ± 0.05	-0.02 ± 0.05
	VLF	0.97 ± 0.05		0.85 ± 0.05	0.89 ± 0.06	-0.07 ± 0.02
	HUF	1.05 ± 0.06		0.93 ± 0.06	0.99 ± 0.05	-0.06 ± 0.02
**HDL Cholesterol^2 ^**mmol/L	VLCARB	1.26 ± 0.05	1.27 ± 0.05	1.26 ± 0.05	1.32 ± 0.05	0.06 ± 0.03^a^
	VLF	1.31 ± 0.07	1.18 ± 0.05	1.15 ± 0.06	1.25 ± 0.06	-0.06 ± 0.04^b^
	HUF	1.26 ± 0.05	1.15 ± 0.05	1.15 ± 0.05	1.19 ± 0.04	-0.06 ± 0.03^b^
**Triacylglycerols^2 ^**mmol/L	VLCARB	1.83 ± 0.19	1.20 ± 0.12	1.16 ± 0.10	1.11 ± 0.10	-0.73 ± 0.12^a^
	VLF	1.51 ± 0.13	1.31 ± 0.10	1.38 ± 0.12	1.44 ± 0.13	-0.06 ± 0.13^b^
	HUF	1.56 ± 0.11	1.27 ± 0.12	1.29 ± 0.11	1.42 ± 0.12	-0.15 ± 0.07^b^
**Glucose **mmol/L	VLCARB	5.3 ± 0.1	5.2 ± 0.1	5.3 ± 0.1	5.3 ± 0.1	-0.1 ± 0.1
	VLF	5.3 ± 0.1	5.1 ± 0.1	5.2 ± 0.1	5.3 ± 0.1	-0.1 ± 0.1
	HUF	5.4 ± 0.1	5.4 ± 0.1	5.2 ± 0.1	5.2 ± 0.1	-0.2 ± 0.1
**Insulin^2 ^**mU/L	VLCARB	10.7 ± 1.1	8.2 ± 0.8	8.1 ± 1.0	7.1 ± 0.8	-3.6 ± 0.5^a^
	VLF	8.6 ± 0.7	8.2 ± 0.7	7.8 ± 0.8	9.9 ± 1.9	1.3 ± 1.7^b,c^
	HUF	9.1 ± 0.6	9.0 ± 0.6	7.9 ± 0.6	7.4 ± 0.7	-1.7 ± 0.5^a,c^
**Folate **nmol/L	VLCARB	23.13 ± 1.46			22.52 ± 1.03	-0.61 ± 0.84
	VLF	23.99 ± 1.42			27.54 ± 1.10	3.54 ± 1.30
	HUF	23.95 ± 1.32			24.83 ± 1.61	0.88 ± 1.82
**Homocysteine^2 ^**umol/L	VLCARB	7.28 ± 0.33			7.76 ± 0.39	0.56 ± 0.27^a^
	VLF	7.30 ± 0.45			6.80 ± 0.34	-0.50 ± 31^b,c^
	HUF	7.14 ± 0.32			7.19 ± 0.30	-0.04 ± 0.22^a,c^
**C reactive protein **mg/L	VLCARB	5.27 ± 0.71		.	4.51 ± 0.60	-0.76 ± 0.56
	VLF	4.52 ± 0.78			3.42 ± 0.70	-1.10 ± 0.50
	HUF	4.52 ± 0.70			4.17 ± 0.71	-0.35 ± 0.71

^1^mean ± SEM

VLCARB = very low carbohydrate (n = 24) VLF = very low fat (n = 22) HUF = high unsaturated fat (n = 21)

^2 ^Significant main effect of diet using repeated measures ANOVA with time as the within subject factor and diet as the between subject factor after adjustment for weight loss as a covariate (P < 0.05)

^3 ^When significant main effect of diet detected, post hoc analysis was conducted using Tukeys test. Variables with the different superscripts are significantly different from one another (P < 0.05)

Diet composition significantly affected the change in HDL-C with or without correcting for weight loss (p = 0.023 unadjusted and p = 0.029 adjusted for weight loss) with an increase on VLCARB (0.06 ± 0.03 mmol/L) whereas the other diets resulted in similar net decreases of 0.06 ± 0.03 mmol/L.

Similarly diet had a significant lowering effect on TG (p = 0.001 unadjusted and p = 0.002 adjusted for weight loss) with VLCARB having the greatest TG reduction (-0.73 ± 0.12 mmol/L) followed by the HUF diet (-0.15 ± 0.07 mmol/L) and VLF the least change (-0.06 ± 0.13 mmol/L).

### Inflammatory markers

Five subjects had C reactive protein (CRP) >15 mg/L at baseline or at the completion of the study and were excluded from the analysis. All diets resulted in a significant decrease in CRP with weight loss, independently of diet (p = 0.037).

### Plasma folate, homocysteine and B12

The main effect of weight loss or diet composition on changes in plasma folate failed to reach statistical significance (p = 0.106 and p = 0.09 respectively) whereas plasma homocysteine increased 6.6% on VLCARB, decreased 6.8% on the VLF and remained unchanged on the HUF diet (P = 0.026 for diet effect) (Table 5). Increases in homocysteine concentrations were observed in 16/24 subjects on VLC, 10/22 on VLF and 10/21 on HUF. There were no changes in plasma B12 levels over the course of the study and levels remained static at 266 ± 13 pmol/L.

### Blood pressure

There was no significant effect of diet composition on blood pressure changes with weight loss resulting in a net reduction in blood pressure of 7 ± 2 mmHg systolic and 3 ± 1 mmHg diastolic blood pressure.

### Glucose and insulin

Fasting glucose decreased with weight loss by 2% (p = 0.036) independently of diet composition (p = 0.10) (Table 5). However, diet composition significantly affected fasting insulin levels (main effect of diet p = 0.004 unadjusted and p = 0.006 adjusted for weight loss) with the VLCARB diet lowering fasting insulin concentrations by 33%, HUF by 19% whereas it increased 15% on VLF (Table 5).

### Post prandial glucose and insulin responses to oral glucose and test meals

**Figure 5:**
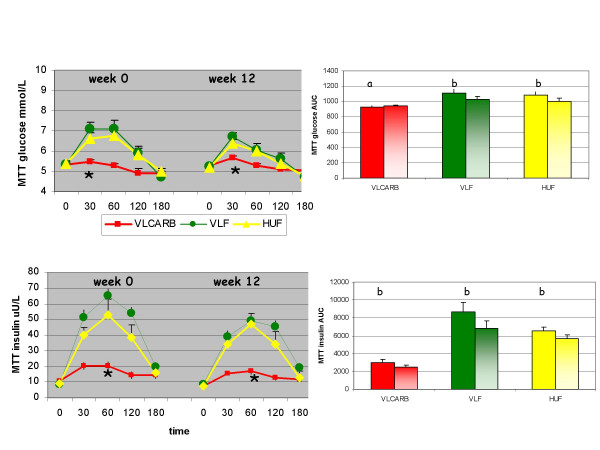
**Plasma glucose and insulin response for 3 h meal tolerance test (MTT) by dietary treatment^1^**. ^1^Mean (± SEM) plasma glucose and insulin concentrations at baseline and 30, 60, 120 and 180 minutes and total AUC after the ingestion of the test meals (Table 2B) at weeks 0 and 12. The main effect of the test meals at week 0 and 12 were compared by repeated-measures ANOVA with week and blood sampling time as within subject factors, and diet as between subject factors. The main effect of time (weight loss) for each diet was compared using repeated-measures ANOVA with AUC at week 1 and 12 as within subject factors. VLCARB = very low carbohydrate diet (n = 24) VLF = very low fat diet (n = 22) HUF = high unsaturated fat (n = 21). * VLCARB test meal significantly different from VLF and HUF test meals at week 0 and week 12, *P *< 0.01. a denotes no significant effect of weight loss within diet group b = significant effect of weight loss within diet group (P < 0.05)

**Figure 6:**
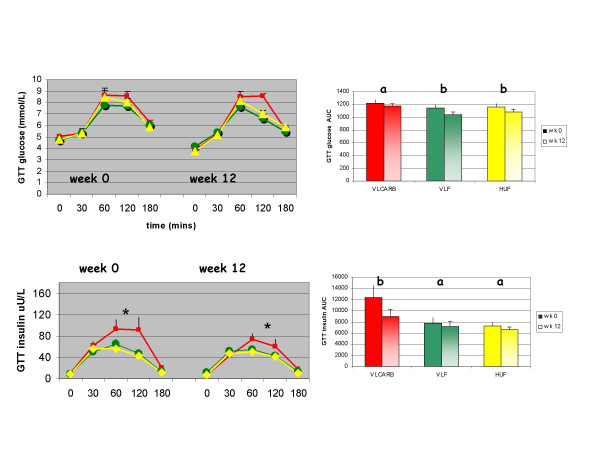
**Plasma glucose and insulin response (mean ± SEM) for 3 h 75 g oral glucose tolerance test (GTT) by dietary treatment^1^**. ^1^Mean (± SEM) plasma glucose and insulin concentrations at baseline and 30, 60, 120 and 180 minutes and total AUC after the ingestion of the 75 g oral glucose drink at weeks 0 and 12. The main effect of diet was compared using repeated-measures ANOVA with week and blood sampling time as within subject factors, and diet as between subject factors. The main effect of time (weight loss) for each diet was compared using repeated-measures ANOVA with AUC at week 1 and 12 as within subject factors. * VLCARB significantly different overall from VLF and HUF at week 0 and week 12, *P *< 0.01. a = no significant effect of weight loss within diet group. b = significant effect of weight loss within diet group (P < 0.05). VLCARB = very low carbohydrate diet (n = 24) VLF = very low fat diet (n = 22) HUF = high unsaturated fat (n = 21).

At baseline, glucose tolerance (as assessed by the total area under the curve) was not different according to diet allocation (P = 0.552) but the insulin response to glucose was significantly different (P = 0.038) with a greater insulin AUC in the VLCARB group compared to the other two groups (P < 0.05). There was a significant effect of diet on the test meal glucose response (P = 0.016) (Figure 5) with the VLCARB meal provoking a lower glucose response than the VLF meal (P = 0.014 post hoc analysis) and the HUF meal (P = 0.054 post hoc analysis). This effect was strengthened if adjustment was made for the differences in baseline insulin AUC as a covariate (P = 0.005). The VLCARB meal also induced an insulin response that was substantially lower compared to HUF and VLF meals (both P < 0.001 on post hoc analysis). Weight loss on the diets resulted in improvements in glucose tolerance in subjects allocated the VLF and HUF diets (P < 0.05) whereas no changes in glucose tolerance were observed in the VLCARB group (Figure 6). However, the insulin response to the glucose challenge was significantly lowered after weight loss on VLCARB (P = 0.016) but the small reductions observed on VLF and HUF diets were not statistically significant. Weight loss provoked a lower insulin response to the test meals (P < 0.05).

### Urinary bone markers and electrolytes

Calcium excretion increased 25% on the VLCARB diet yet decreased 12–16% on both the VLF and HUF (Table 6). Sodium excretion was not significantly different by time or diet whereas potassium excretion increased only on VLF (P < 0.001). Markers of bone turnover Dpr:Cr and Pyr:Cr increased significantly overall by 15% with weight loss (P < 0.001), but no specific diet composition effects were observed.

**Table 6:** 24 hour urinary excretion of Calcium, Sodium, Potassium and Crosslinks before and after weight loss^1^

	**VLCARB**		**VLF**		**HUF**	
	**Baseline**	**12 weeks**	**Baseline**	**12 Weeks**	**Baseline**	**12 Weeks**
Calcium^2 ^mmol/24 hr	4.9 ± 0.5	6.1 ± 0.6^a^	4.3 ± 0.4	3.6 ± 0.4^b,c^	4.3 ± 0.6	3.8 ± 0.5^a,c^
Sodium^3 ^mmol/24 hr	172.7 ± 16.2	163.6 ± 15.2	173.9 ± 10.6	168.3 ± 13.1	175.4 ± 16.5	141.6 ± 11.9
Potassium^4 ^mmol/24 hr	80.0 ± 4.8	65.2 ± 3.9^a^	83.2 ± 5.7	98.1 ± 5.8^b,c^	77.8 ± 5.9	74.3 ± 5.0^a,c^
Deoxypyridinoline:creatinine^5 ^nmol/mmol	18.0 ± 1.0	20.5 ± 1.6	21.2 ± 1.5	23.1 ± 1.5	19.4 ± 1.1	22.4 ± 1.2
Pyridinoline:creatinine^5 ^nmol/mmol	64.5 ± 3.0	73.3 ± 4.2	71.9 ± 5.3	83.9 ± 5.7	67.0 ± 2.9	77.1 ± 3.0

^1 ^Mean ± SEM

^2 ^Main effect of diet p = 0.025 using repeated measures ANOVA. Variables with different superscripts are significantly different using Tukey's test for post hoc analysis (P < 0.05).

^3 ^No significant effect of time or diet using repeated measures ANOVA

^4 ^Main effect of diet P < 0.001. Variables with different superscripts are significantly different using Tukey's test for post hoc analysis (P < 0.05).

^5 ^Main effect of time using repeated measures ANOVA P < 0.0001

VLCARB = very low carbohydrate diet (n = 24) VLF = very low fat diet (n = 22) HUF = high unsaturated fat (n = 21)

## Discussion

This study has attempted to evaluate isocaloric dietary patterns that are very low and high in carbohydrate. Although the diets were consumed under free-living conditions and nutrient intakes analyzed using food records, considerable effort was taken to ensure that this was achieved by the provision of key foods and providing very prescriptive diet information and menu plans. We noted that percentage weight loss was greater on the VLCARB diet compared to the VLF diet, providing possible evidence of a metabolic advantage. We have shown that the amount of weight loss on a VLCARB diet is greater than similarly energy restricted higher carbohydrate patterns as has been observed some decades ago in albeit lower kilojoule but isocaloric comparisons [18,20]. Our observation that this difference was due primarily to loss of lean mass is consistent with the findings of Vasquez & Adibi [19] but no isocaloric dietary studies such as ours have been conducted to confirm these findings. As previously shown, the amount of fat loss was similar on all diets when the same energy restriction is applied. It is surprising that despite a higher kilojoule intake than prior studies with a consequently smaller energy restriction as well as a longer study duration, we still noted a greater effect on lean mass loss for the VLCARB pattern. Other authors have argued that, by reducing plasma insulin levels, a low-carbohydrate, ketogenic diet would spare body protein by minimizing the need for gluconeogenesis [21]. Although we and others [22] did indeed observe greater reductions in both fasting and post prandial insulin responses on VLCARB, this was not associated with protein sparing. Volek et al [23] observed an increase in lean mass in a small study in normal weight men on a VLCARB in energy balance but a subsequent study by the same author in overweight subjects using VLCARB in energy restriction showed no greater lean mass preservation [24].

One of the key findings of this study was that fasting and post prandial insulin was lower on the VLCARB diet than the other two high carbohydrate diets. We believe that the provision of a glucose tolerance test as well as a "meal test" was a major strength of this study. The virtual flat line glucose and insulin response to a low-carbohydrate meal in the VLCARB group (Figure 5) is remarkable data that clearly shows how effective this dietary pattern is at stabilizing the metabolic and hormonal milieu that is the goal for people with insulin resistance and type II diabetes. The fact that the low-carbohydrate diet did not worsen, and even improved, the glucose and insulin response to 75 g of glucose further emphasizes the fact that carbohydrate-restricted diets improve insulin sensitivity provided they achieve weight loss. Dysregulation of insulin function and glucose metabolism is the hallmark of diabetes and the fact that a low-carbohydrate diet can significantly improve this aspect of metabolism is noteworthy.

Our study is unique in that, unlike previous studies using whole foods that have used an *ad libitum *approach in implementing the dietary strategy, we have attempted to control and match total kilojoule intake on all diets as well as introducing an energy balance period to separate the effects of diet composition and weight loss from energy restriction. Consequently we observed an increase in LDL cholesterol on VLCARB compared to a reduction on the two low saturated fat dietary patterns. This is in contrast to several previous studies [7,13] who saw no increase in LDL cholesterol from baseline levels on VLCARB. This is most likely due to the lowering effect of weight loss on LDL cholesterol attenuating the expected rise from an increase in saturated fat intake. This effect of weight loss on LDL cholesterol has been estimated to be a reduction of 0.02 mmol/L per kilogram weight loss [25] whereas the increase in LDL cholesterol for every 1% energy increase in saturated fatty acids is estimated to be 0.03 mmol/L [26]. Therefore the likely net effect on LDL cholesterol with weight loss on VLCARB will be a balance between how much weight is lost versus the increase in saturated fatty acids. For small weight losses the impact is likely to represent a net increase in LDL cholesterol whereas for moderate weight losses this effect may be neutral. However, the cardiovascular risk represented by these changes in LDL cholesterol are not clear as Sharman showed that more men with "*pattern B*" had switched to "*pattern A*" after 6 wk of intake of a very low-carbohydrate diet (75%) compared with a low-fat diet (42%) [27]. An examination of the effect on apoB concentrations revealed no significant effect of diet composition on repeated measures ANOVA although both VLF and HUF resulted in a net lowering of apoB concentrations (P < 0.05 Students paired t test) whereas for VLCARB it remained unchanged from baseline despite a significant fall during active weight loss. This represents a balance between the rise in cholesterol-rich particles and the fall in triacylglycerol-rich particles with this diet. Volek et al have proposed a model of lipoprotein metabolism on VLCARB diets that is consistent with the observed decrease in triacylglycerols concentrations, increase in HDL-Cholesterol, and a redistribution of LDL to a larger particle size [28]. We did observe a significant effect of gender to the apoB response to refeeding to weight maintenance (P < 0.05). This has not previously been described and suggests that in men, apoB may be more resistant to caloric flux than in women. ApoB may arguably be a better predictor of vascular risk [29] although there is some controversy in this area. When triacylglycerol is elevated, such as in people with type 2 diabetics, apoB (or non HDL cholesterol) is clearly superior but this may not be true in people with normal triacylglycerol levels. We also observed a diet effect on apoB48 which was unexpected and may be related to high fat diets increasing chylomicron clearance.

The greater triacylglycerol reduction on VLCARB is in keeping with what is anticipated on isocaloric lower carbohydrate patterns and also consistent with what has been observed in longer term *ad libitum *studies when adjusted for weight loss [11,13,26,30]. Low HDL-C and hypertriglyceridemia have been shown to be independently related to the risk of myocardial infarction [31,33]. In the Veterans Affairs High – Density Lipoprotein Intervention Trial [33] which used gemfibrozil, it was observed that for every 1% increase in HDL-C, there was a 3% reduction in death or myocardial infarction although not all of this effect was attributed to the effect on HDL-C. It is therefore, possible that weight loss on dietary patterns that are very low in carbohydrate and which improve these risk factors may be therapeutic for subjects with this pattern of dyslipidaemia despite much of the fat being saturated. However there is currently minimal evidence that increasing HDL cholesterol with fat is protective.

CRP is an independent risk factor for cardiovascular disease and abdominal obesity is associated with elevations of CRP [34]. In the present study weight loss per se resulted in a reduction in CRP irrespective of dietary macronutrient composition. Reduction in CRP with weight loss has been observed previously by us in a study using very-low-fat diets and in a recent study when CRP fell irrespective of dietary macronutrient composition [35,36]. Others al have also reported reductions in CRP on VLCARB [37,38]. We also observed a beneficial effect on blood pressure with weight loss which was independent of diet composition. Other authors have reported blood pressure reductions with modest weight loss [39]. There is known to be a strong positive association between systolic blood pressure and increasing risk of stroke and cardiovascular disease, and reductions in systolic blood pressure contributes to overall CVD risk reduction [40].

The very low insulin response observed following the VLCARB test meal was unexpected given that protein is well known to stimulate insulin secretion[41]. However it is likely that the large amount of fat in the test meal markedly delayed gastric emptying of the protein and blunted the rise in insulin [42]. Although apparent glucose tolerance did not change with weight loss on VLCARB, the insulin response to both glucose and the test meals was lowered suggesting improvements in insulin sensitivity.

The other cardiovascular risk factor which worsened on VLCARB was plasma homocysteine which increased by 6.6% despite no differences in plasma folate. This small increase in homocysteine may or may not have clinical significance as homocysteine levels after the VLCARB diet were low at 7.76 umol/L, which are below values that are indicative of higher risk for cardiovascular disease. Hyperhomocysteinemia was found to be an independent risk factor for cardiovascular disease in a prospective study of plasma homocysteine and risk of myocardial infarction in US physicians [43] although in men free of coronary disease high circulating homocysteine concentrations were not a risk factor for acute coronary events. The same study showed that folate concentrations are inversely associated with the incidence of acute coronary events. In our study, folate concentrations were measured during energy balance during which time absolute carbohydrate intake and vegetable intake were higher than during the energy restriction period. Hence it is possible that plasma folate levels were lower during the energy restriction period suggesting a need for folate supplementation on VLCARB dietary patterns. Although mandatory folate fortification does not occur in Australia, it is likely that such fortification which does occur in countries such as the US may minimize these effects.

We noted an increase in calcium excretion on VLCARB. This is in contrast to a reported previous report from our group which showed that while weight loss was associated with increased bone resorption on a higher protein diet (34% energy from protein) calcium excretion decreased [44]. Metabolism of dietary protein (particularly fish, meat, and cheese) is associated with acid generation, which can reduce blood pH and cause obligatory calcium losses whereas metabolism of fruit, and vegetables (both of which were low in VLCARB) produces alkali, which can partially ameliorate the effect of this acid [45,46]. On the other hand, a protein intake greater than 87 g/day is related to improved lower limb bone mass in elderly women. Calcium intake on VLCARB was significantly higher than on VLF and not different to HUF. Hence the possible adverse effects of long term use of VLCARB dietary patterns on bone mass remains speculative.

## Conclusion

Under isocaloric conditions VLCARB results in similar fat loss to other conventional dietary patterns although the greater percent weight loss is suggestive of a metabolic advantage. VLCARB resulted in equal improvements in most cardiovascular risk factors compared to conventional weight loss diets while the triacylglycerol reduction offset the LDL cholesterol rise. The more favorable effects of VLCARB on fasting and post prandial plasma insulin concentrations is a significant observation which indicates that this dietary pattern may be a useful strategy for the short-term management of subjects with insulin resistance and hypertriacylglycerolemia.

## Competing interests

The author(s) declare that they have no competing interests.

## Authors' contributions

Manny Noakes and Peter Clifton designed the study, performed statistical analysis and wrote the manuscript. Paul Foster and Jennifer Keogh contributed both to the analysis, interpretation of the data and preparation of the manuscript and were involved in the dietetic counseling and conduct of the study. Tony James and John Mamo performed the ApoB48 analyses.
